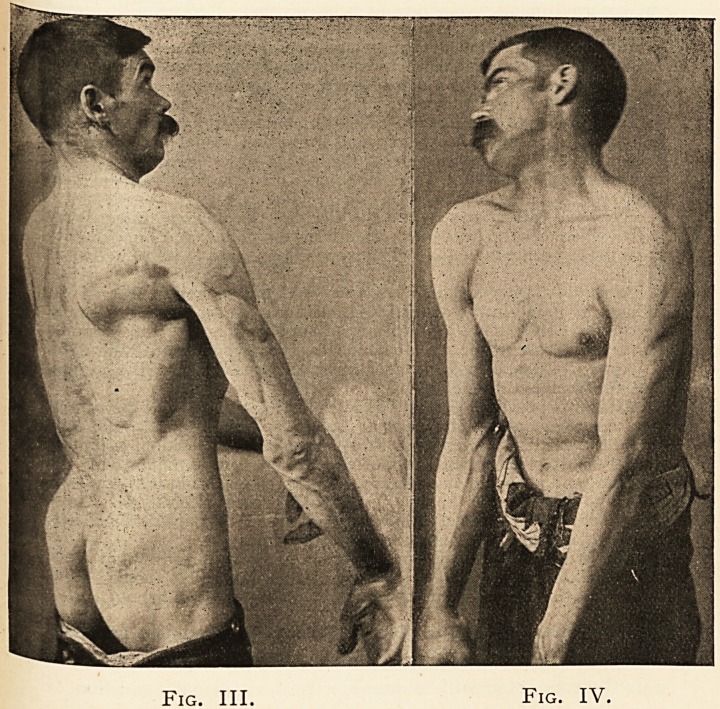# A Case of Double Spasmodic Torticollis: Excision of Both Spinal Accessory Nerves

**Published:** 1896-12

**Authors:** R. Shingleton Smith

**Affiliations:** Emeritus Professor of Medicine, University College, Bristol; Physician to the Bristol Royal Infirmary


### A CASE OF DOUBLE SPASMODIC TORTICOLLIS: EXCISION OF BOTH SPINAL ACCESSORY NERVES.

BY

R. Shingleton Smith, M.D., B.Sc. Lond., F.R.C.P. Lond.,

*Emeritus Professor of Medicine, University College, Bristol;*
*Physician to the Bristol Royal Infirmary.*

Dr. Maurice H. Richardson and Dr. George L. Walton, in an excellent paper[1] on the operative treatment of spasmodic torticollis, give the following summary of conclusions:—

1. Palliative treatment, whether by drugs, apparatus, or electricity, will rarely prove successful in well-established spasmodic torticollis.

[1] *Am. J. M. Sc.*, 1895, cix. 27.

2. Massage may prove of value in comparatively recent cases.

3. Resection affords practically the only rational remedy.

4. Operation on the spinal accessory nerve may afford relief, even if other muscles than the sterno-cleido-mastoid are affected. On the other hand, the affection previously limited to the sterno-cleido-mastoid may spread to other muscles, in spite of this operation.

5. No fear of disabling paralysis need deter us from recommending operation, as the head can be held erect even after the most extensive resection.

6. The most common combination of spasm is that involving the sterno-mastoid on one side and the posterior rotators on the other, the head being held in the position of sterno-mastoid spasm with the addition of retraction through the greater power of the posterior rotators.

7. It seems advisable in most cases to give preference to the resection of the spinal accessory as the preliminary procedure.

The following case is an extreme illustration of this intractable disorder, and it was not likely that any kind of surgical interference would subdue a long-standing habit of twenty years. Nevertheless the result, although not as successful as one hoped for, more than justifies the operative measures adopted :—

J. P., aged 30, single, a collier, living at Aberbeeg, Mon., was admitted to the Bristol Royal Infirmary on January 27, 1896, in consequence of long-continued muscular spasms of the head and limbs.

*Family History.*—His father died at 59 from chronic bronchitis. His mother is alive and in good health. Four brothers and three sisters are well, with no evidence of rheumatism in family.

*Personal History.*—He was a healthy boy up to the age of 10. When at school his head was twisted forcibly towards the left shoulder, and then became fixed. When he tried to get the head straight he was unable to do so, and it remained in this position, drawn to left side, for some months. Six months afterwards he had scarlet fever, was seriously ill, and lost his voice, which has never quite regained the normal. One month after the onset of the attack he began to have some kind of tremor, but the stiffness of the neck was the only thing present up to the time of the fever. These spasms, tremors, and involuntary movements of various kinds have continued with varying severity ever since, and have always caused him some difficulty in walking. His speech has been affected at times, and he has been unable to speak at all for two or three hours together, but he has not been absolutely laid up, and he has always been able to dress and feed himself without assistance. There is no history of any specific disease, and there is no evidence of anything of the kind. During the last twelve months the spasmodic movements have been getting worse, and the difficulty in walking has increased.

On admission patient has the appearance of a well-nourished man, of short stature—5 ft. 6 in.—but of great muscular development, weighing ten stone. His general health appears to be good, but he cannot control his hypertrophied muscles, and hence he is not able to stand still, but his head and arms are subject to oft-repeated involuntary muscular spasms, somewhat of a choreic type, and the arm movements simulate those of athetosis. Whilst sitting quietly these movements are not active, but when he gets excited, and more particularly when he walks, the arms and head are thrown into a condition of involuntary spasm involving a great number of muscles, distributed equally on both sides, but acting intermittingly, although more slowly than the ordinary movements of chorea. As regards the head, the movement simulates that of spasmodic torticollis, first of one side and then of the other, then of both, the head being strongly flexed on the chest and then drawn back by the posterior cervical muscles. At the same time one or the other shoulder is drawn up, and the head drawn over to one side or the other.

When lying quietly in bed the head is fairly still, and the movements cease entirely during sleep.

The most striking feature in the movement is the powerful contraction of the sterno-mastoids and trapezii, and at the same time the platysma myoides is seen to stand out strongly.

All the muscles concerned in the movements are greatly hypertrophied; the sterno-mastoids appear to be two or three times their normal size, and hence the neck was found to measure 16½ inches in circumference. The hypertrophy is not limited to the neck; the thoracic and abdominal muscles stand out remarkably, with very well defined transverse lines. Sometimes, when violent attacks of spasm come on, the patient feels some difficulty in breathing. The muscles of arms and forearms are also in excess, but the dynamometer does not show any great increase in power, the left hand giving a pressure of 75 pounds, whilst the right gives 100. Of the facial muscles the occipito-frontalis is often strongly affected, but the tongue is normal. The patient states that the violent spasms commence at the abdomen, the wall of the abdomen becoming like a board, whilst the spasm passes upwards to the head and neck. Walking is accomplished with some difficulty. He struggles forwards "as if against a strong force holding him back," but when he walks backwards there is no such difficulty, as the spasm is less.

Fibrillary movements are visible in some muscles, different strands of fibres acting more or less independently. There are no sensory defects. The reflexes are not remarkably abnormal, but the superficial are ill-marked, the cremasteric and abdominal being absent. The knee-jerk is in excess on the right side, but there is no ankle- or rectus-clonus. The electrical reactions, motor and sensory, are quite normal: the optic discs are normal, and there is no evidence of any eye defect.

This patient's symptoms were so obviously beyond the range of medicinal treatment that little was done in this way. The only drug found to be useful was hyoscine hydrobromate, which not only gave a good night but appeared to diminish the violence of the movements during the day following. Arsenic, belladonna, and Calabar bean were given in increasing doses, but without favourable result.

After repeated consultations with my surgical colleague, Mr. W. H.

Harsant, and as it seemed clear to us that nothing short of the most decided surgical interference gave any prospect of improvement, it was decided that excision of both spinal accessory nerves should be performed, and this was done by Mr. Harsant on the first of

FIG. I. FIG. II.

[Figs. I. and II. show fairly characteristic positions of the patient previous to operation. It will be seen that the head is firmly fixed with both sterno-mastoid muscles in rigid spasm, and that the platysma is also in violent contraction. The occipito-frontalis and orbicularis palpebrarum are also in action. The arms are rigid, and the abdominal recti are in powerful action.

Fig. II. shows also the elevation of the shoulder and the lateral bending of the head. It also shows the great development of the erector spinæ and the other dorsal muscles.]

April. Gas and ether having been given, all the involuntary spasmodic movements ceased, and the head assumed the normal position. The neck having previously been wrapped round with carbolic pads, and afterwards washed with perchloride of mercury solution, an incision

FIG. III. FIG. IV.

[Figs. III. and IV. show the difference in position six weeks after the operation. It will be seen that there is now more retraction of the head and less elevation of the shoulders. The spasm of the arms, of the dorsal muscles (even the glutei) and of the occipito-frontalis is as marked as before. The scars of the operations are visible, and the left sterno-mastoid muscle is seen to be still in a condition which may be described as the antithesis of paralysis.]

was made on the right side along the line of the anterior border of the sterno-mastoid, and about one-eighth of an inch anterior to it, extending from the level of the mastoid process to that of the angle of the jaw. The tissues between the anterior border of the sterno-mastoid and the posterior belly of the digastric muscles were carefully dissected, the external jugular vein having been divided after being ligatured above and below. As the nerve could not be found the incision was extended in a direction at a right angle, and the sterno-mastoid was partially divided transversely. By turning up the upper cut end the trunk of the nerve was then found, and a piece of nerve of three-quarters of an inch in length was removed. The divided muscle was sutured with catgut, the wound was closed by a continuous suture, and dressed with iodoform gauze and absorbent wool. The left spinal accessory was then sought for, the incision being made at a lower level than before. It was easily found, and a similar length (three-quarters of an inch) removed.

April 2.—Patient was a little steadier, but the movements were very active whenever the head was raised from the pillow, and he wished to have the hyoscine injection. The trapezii appeared to be as active as before.

April 5.—The spasmodic contractions were so active that it was difficult to maintain the dressings in position. Accordingly the wound on the right side showed indications of suppuration, and the patient's temperature rose to 102.2°. Morphia was given hypodermically at night.

April 7.—A small collection of pus was released from the wound, and the temperature fell to 98°.

After this period the wounds gave no further trouble, and the patient was able to get about the ward as before, and in a few days to walk round the garden. There was little change in his condition, excepting that the posterior cervical muscles appeared to predominate over the anterior, and accordingly in any violent attack of spasm the head was drawn backward, with the face turned up more than formerly. There was no actual paralysis of either sterno-mastoid or trapezius muscles, which all appeared to contract violently at times.

His writing on the 19th of May showed great improvement since the operation. He left the Infirmary at this date, as it seemed clear that no further operative interference was likely to be of benefit.

This case was from the first clearly beyond the range of medicinal control. Overactive and hypertrophied muscles must be connected with a more or less abnormal condition of the nerve-centres with which they are physiologically connected, and experience has shown that it is practically impossible to control this kind of rebellion of muscle and nerve when the overactivity has become a long-standing habit.

Although medicinal aids have shown themselves to be ineffective, yet the effects of surgical severance of the connection between the muscles and their nerve-centres have been so encouraging as to render operative interference imperative in cases where the spasm is limited to the area of particular nerves.

But further, Noble Smith has shown[1] that even in cases where the spasm has extended far beyond the ordinary range of torticollis, yet the removal of the focus from which the spasm has commenced gives rise to satisfactory results over a much wider area. He endeavours to show "that section and ablation of a piece of the spinal accessory nerve is absolutely certain to remove all spasm from the muscles supplied by that nerve, and is very likely to remove spasms set up in other muscles, although other nerves are apparently involved," and "that the operations of section of the spinal accessory nerve, and of the posterior roots of the cervical nerves, are not followed by serious inconvenience to the patient from paralysis of the muscles."

Drs. Richardson and Walton remark[2]: "It may now be fairly assumed . . . that treatment by drugs is useless, that serious cases cannot bear the restraint of apparatus, and that electricity in all forms is ineffective." They hope for better results from rest in bed, hypnotism, and massage; and they go on to observe that "few sufferers appeal more strongly to our sympathies than the class under consideration. Those who have seen cases of moderate severity only, or cases rheumatic in character, can hardly appreciate the pain and distress caused by spasmodic wry-neck of the severe type. Such patients are rendered unfit for occupation or society, resting the head either in the recumbent or semi-recumbent position, or unsuccessfully attempting to relieve the spasm by holding the hand to the cheek. Before many years have elapsed they, as a rule, welcome operation, even though only moderate hope of cure is offered."

The principal question for solution now is, not whether operation on the spinal accessory nerves is justifiable, but how much farther is it wise to go? Resection of the external branches of the posterior divisions of the second and third cervical nerves supplying the splenius muscle, and of the sub-occipital supplying the rectus capitis posticus major, has been frequently performed by Noble Smith, Powers, Gardner, Keen, and others, and the results have justified the attempts. Keen reports a satisfactory result in three out of four cases.[3] It

[1] *Spasmodic Wry-neck*, 1891. [2] *Am. J. M. Sc.*, 1896, cxii. 39, 40.
[3] *Text-Book on Nervous Diseases*. Edited by Francis X. Dercum, M.D. 1895.

appears that the excision of these nerves on both sides does not impair the ability to hold the head erect, and on the other hand the spasm is greatly reduced or made to disappear, sometimes by section of the spinal accessory nerves only, or by subsequent section of the posterior branches of the cervical nerves.

In the case now reported the habit of overaction had been in existence for twenty years, and the hypertrophy was so great and so widely distributed beyond the area of the spinal accessory nerves, that there was little prospect of relief. However, the ablation of the two nerves has produced a decided improvement in the condition of the patient, although it has not abolished the tendency to spasm over a much wider area than could be controlled by the spinal accessory nerves.

## Figures and Tables

**Fig. I. Fig. II. f1:**
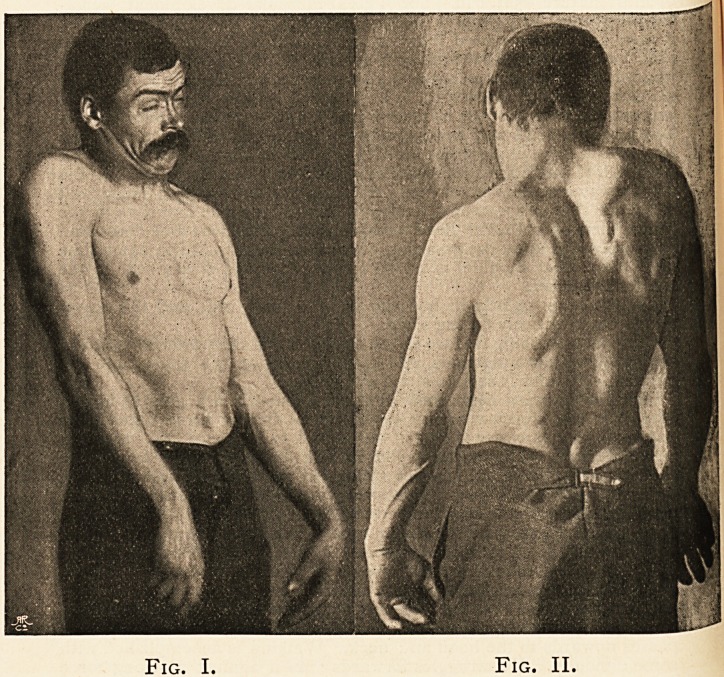


**Fig. III. Fig. IV. f2:**